# Strain Conditions for the Inverse Heusler Type Fully Compensated Spin-Gapless Semiconductor Ti_2_MnAl: A First-Principles Study

**DOI:** 10.3390/ma11112091

**Published:** 2018-10-25

**Authors:** Tie Yang, Liyu Hao, Rabah Khenata, Xiaotian Wang

**Affiliations:** 1 School of Physical Science and Technology, Southwest University, Chongqing 400715, China; h998200@126.com; 2 Laboratoire de Physique Quantique de la Matière et de Modélisation Mathématique, Université de Mascara, Mascara 29000, Algeria; khenata_rabah@yahoo.fr

**Keywords:** Heusler compound, density functional theory, first-principles calculation, Spin-gapless semiconductor

## Abstract

In this work, we systematically studied the structural, electronic, magnetic, mechanical and thermodynamic properties of the fully compensated spin-gapless inverse Heusler Ti_2_MnAl compound under pressure strain condition by applying the first-principles calculation based on density functional theory and the quasi-harmonic Debye model. The obtained structural, electronic and magnetic behaviors without pressure are well consistent with previous studies. It is found that the spin-gapless characteristic is destroyed at 20 GPa and then restored with further increase in pressure. While, the fully compensated ferromagnetism shows a better resistance against the pressure up to 30 GPa and then becomes to non-magnetism at higher pressure. Tetragonal distortion has also been investigated and it is found the spin-gapless property is only destroyed when c/a is less than 1 at 95% volume. Three independent elastic constants and various moduli have been calculated and they all show increasing tendency with pressure increase. Additionally, the pressure effects on the thermodynamic properties under different temperature have been studied, including the normalized volume, thermal expansion coefficient, heat capacity at constant volume, Grüneisen constant and Debye temperature. Overall, this theoretical study presents a detailed analysis of the physical properties’ variation under strain condition from different aspects on Ti_2_MnAl and, thus, can provide a helpful reference for the future work and even inspire some new studies and lead to some insight on the application of this material.

## 1. Introduction

Half-metallic magnets became of great interest and have been widely and largely studied during the last decade because of their potential applications in a variety of novel spintronic devices [1,2,3]. They are characterized by the coexistence of metallic and semiconductoring behaviors in different spin channels, which leads to 100% spin polarization at the Fermi energy level [4,5]. As one of the most promising classes of such materials, Heusler alloys received a lot of attention thanks to the high Curie temperatures [6] and structural similarity with zinc-blende semiconductor [7,8,9]. Heusler alloys are a huge group of ternary and quaternary compounds with more than thousand members. For a ternary Heusler, it is commonly represented by a generic formula as XYZ for half-Heusler and X_2_YZ for full-Heusler, where X and Y are transition metal atoms and Z sp element atom. Thus, different Heusler compounds can be easily designed and tuned by simply varying the element in the periodic table [10,11]. Under normal condition, Heusler compounds crystallize in a highly ordered cubic structure either in Cu_2_MnAl-type or Hg_2_CuTi-type.

In the past ten years, another new class of materials was proposed as spin-gapless semiconductor [12]. It is partially like the above-mentioned half-metals in terms of the band structure: on spin direction with band gap as semiconductor, similar with half-metal, and the other spin direction with zero band gap, i.e., the conduction band minimum and the valence band maximum touch each other exactly at the Fermi level. Later on, several studies have been done and proved that some Heusler compounds also show this spin-gapless semiconductoring behavior [10,13,14,15,16,17], such as Ti_2_MnAl, Mn_2_CoAl, Ti_2_CoSi, Ti_2_VAs and so on. Normally, half-metallic Heusler compounds have net magnetic moment and it follows the Slater-Pauling rule in different forms dependent on the specific material [18,19,20,21]. In particular, some Heusler materials have zero net magnetic moment, such as Ti_2_MnAl, and this is from the compensating effect between the constitute atoms. Thus, Heusler of this feature is called as the fully compensated ferromagnets and they have special advantages and could be more useful due to the following properties: (a) high magnetic transition temperature; (b) inertial to exterior magnetic field; (c) less energy consumption because of zero stray field.

In combination, Ti_2_MnAl has both spin-gapless semiconductoring feature and also fully compensated ferromagnetic property. Consequent, it arouses a lot of interests in research. Such as, Skaftouros et al. [22] first demonstrated the spin-gapless band structure of Ti_2_MnAl from first-principles calculation; Fang et al. [22] analyzed the possible state hybridization and band gap formation in the bulk form and also studied the surface effect from different atomic terminations on the half-metallicity of Ti_2_MnAl; Jakobsson et al. [23] applied the frozen-magnon method together with first-principles calculation to calculate the exchange interactions and spin-wave dispersion to further elucidate the magnetic properties of Ti_2_MnAl; Feng et al. [24] succeeded in growing Ti_2_MnAl thin film on Si substrate by magnetron sputtering and revealed its spin-gapless behavior and nearly compensated ferrimagnetic property; Lukashev et al. [13] studied the electronic property and half-metallic behavior of Ti_2_MnAl under different disorder and also with substitution; Singh et al. [25] studied the variation of the spin-gapless property of Ti_2_MnAl under uniform and tetragonal strain; Han et al. [26] investigate the bias-voltage and temperature-driven spin transport properties of Ti_2_MnAl-based magnetic tunnel junction. Moreover, Shi et al. [27] and Noky et al. [28] recently found the magnetic Weyl semimetal with strong anomalous Hall effect in Ti_2_MnAl.

In this paper, for the purpose to better understand the variation of the physical properties of Ti_2_MnAl under strain condition, we present a study on the pressure dependence of the structural, electronic, magnetic, mechanical and thermodynamic properties based on first-principles calculation and the quasi-harmonic Debye model. The studied pressure ranges from 0 to 50 GPa. Different equilibrium lattice under pressure will be revealed to correlate them together. Besides, tetragonal distortion is also included and its impact is discussed. Several thermodynamic parameters have been calculated and they can provide an exhaustive data as a reference for the future studies.

## 2. Computational Details

### 2.1. Crystal Structure and Equilibrium Lattice

**Figure 1:**
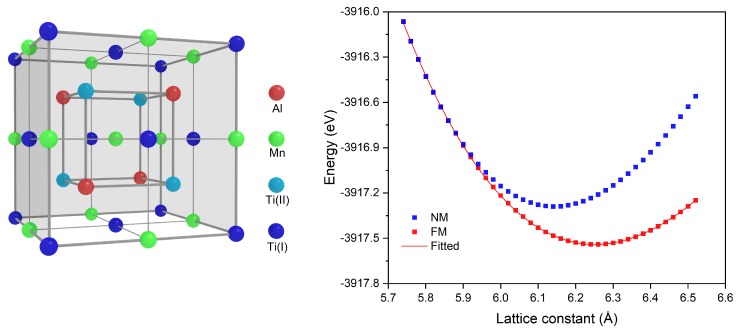
The crystal structure of Ti_2_MnAl compound and its calculated total energy with respect to different lattice constant. The non-magnetic (NM) and ferromagnetic (FM) states are considered.

The Heusler compound normally has a cubic structure with four interpenetrating face-centered-cubic (FCC) sublattices, defined by the Wyckoff coordinates as A(0,0,0), B(1/4,1/4,1/4), C(1/2,1/2,1/2) and D(3/4,3/4,3/4). For Ti_2_MnAl, the Hg_2_CuTi-type structure or the inverse structure was adopted in the present work as it is proved Ti_2_MnAl of this structure has spin-gapless behavior. The Ti(I), Ti(II), Mn and Al atoms occupy A, B, C and D Wyckoff positions respectively, see the corresponding crystal structure in Figure 1. To study the structural, electronic and magnetic properties of the Ti_2_MnAl compound, we have done the first-principles calculation with the pseudo-potential plane-wave method based on density functional theory [29] by using the CASTEP code [30]. The Perdew-Burke-Ernzerhof (PBE) functional within the generalized gradient approximation (GGA) [31] and the ultrasoft pseudo potential are selected for the exchange-correlation potential and the interaction between the atomic core and the valence electrons, respectively. The plane-wave cutoff energy is set as 500 eV for all cases after the initial convergence test. A specific *k*-point mesh is selected using a 15 × 15 × 15 Monkhorst-Pack grid for the integration in the Brillouin zone. The self-consistent field tolerance for the calculations was set as a total energy difference smaller than 5 $\times 10^{-6}$ eV/atom.

For the determination of the equilibrium lattice constant of Ti_2_MnAl, we took account of the non-magnetic and the ferromagnetic states and calculated the total energy against different lattice constants. Results are shown in shown in Figure 1. It clearly displays that the total energy of the ferromagnetic state has lower energy and thus it is energetically more favorable than the non-magnetic state. By polynomial fitting and minimization of the total energy under ferromagnetic state, the obtained equilibrium lattice constant is 6.22 Å and this value matches very well with other studies [32].

### 2.2. Electronic and Magnetic Properties

**Figure 2:**
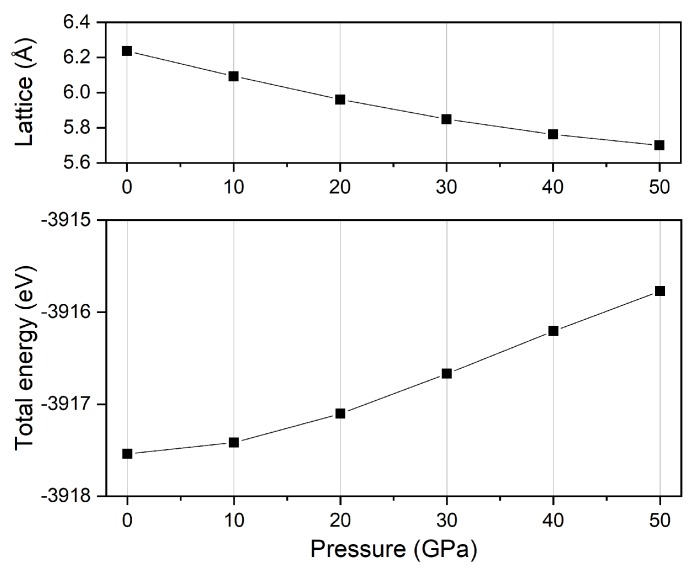
The calculated equilibrium lattice and total energy of Ti_2_MnAl compound at different pressure.

After the equilibrium lattice of Ti_2_MnAl was obtained, we then calculated its electronic structure and magnetic properties with the ultrasoft pseudopotential and PBE-GGA to handle the interactions between electrons and ions and the exchange-correlation functional. Moreover, the structural optimization was carried out for the external pressure application, by using the Broyden-Fletcher-Goldfarb-Shanno minimization method [33]. The following converge parameters were selected for this structure optimization process: the energy difference is within 5 $\times 10^{-6}$ eV/atom; the force change per atom is less than 0.01 eV/Å and the stress of all different components is less than 0.02 GPa. It is shown in Figure 2 that the calculated lattice constants and the total energies vary under different pressure values. As expected, the lattice decreases and the total energy increase with pressure increase. Furthermore, we can find the changing amplitude of the lattice constant is larger at the initial low pressure range (0–20 GPa) and decreases at higher pressure range (30–50 GPa); while, the opposite effect is observed for the total energy. This means that with pressure increase, the Ti_2_MnAl material is compressed less and becomes harder to be further compressed. After obtaining the equilibrium lattice constants at different pressure, we continued to calculate the electronic and magnetic behaviors accordingly under different pressure.

### 2.3. Mechanical Properties

By applying the stress-strain method [34], we can calculate the elastic constants of Ti_2_MnAl compound and thus study its mechanical properties. For a simple structure of cube, the many elastic constants can be reduced into three independent ones, i.e., C_11_, C_12_ and C_14_. The first one reflects the elasticity of the material in length and the other two represent the shape elasticity. Moreover, we can also calculate several other mechanical parameters with these elastic constants by the Voigt-Reuss-Hill approximation [35], including the bulk modulus (B) and the shear modulus (G) as follows:

(1) $$B=\frac{B_{V}+B_{R}}{2},\;G=\frac{G_{V}+G_{R}}{2}$$

where B_V_ (B_R_) and G_V_ (G_R_) are the lower (upper) limit of the Voigt (Reuss) boundary. For a simple cubic crystal structure, they associate with the three independent elastic constants [36]:

(2) $$B_{V}=B_{R}=\frac{C_{11}+2C_{12}}{3},\;G_{V}=\frac{C_{11}-C_{12}+3C_{44}}{5},\;G_{R}=\frac{5C_{44}\left(C_{11}-C_{11}\right)}{4C_{44}+3\left(C_{11}-C_{12}\right)}$$

Immediately, we can also calculate the Young’s modulus (E) and Poisson’s ratio (δ) from B and G with the following formula [37]:

(3) $$E=\frac{9GB}{3B+G},\;\delta =\frac{3B-2G}{2(3B+G)}$$

### 2.4. Thermodynamic Properties

Besides, we also studied the thermodynamic properties of Ti_2_MnAl compound in terms of its dependencies on pressure and temperature by the quasi-harmonic Debye model [38,39,40], which is very useful to understand its particular behavior under severe temperature or pressure constrains. In the framework of the quasi-harmonic Debye model, the non-equilibrium free energy can be described by the following formula

(4) $$G\left(V;\;P,\;T\right)=E\left(V\right)+PV+A_{VIB}\left(\Theta_{D}\left(V\right);\;T\right)$$

where E(V) is the total energy per formula unit of Ti_2_MnAl, PV describes the constant hydrostatic pressure condition, Θ_D_(V) is the Debye temperature and A_VIB_ represents the vibrational Helmholtz free energy, which is given by the following expression with the phonon density

(5) $$A_{VIB}\left(\Theta_{D};\;T\right)=nk_{B}T\left[\frac{9}{8}\frac{\Theta_{D}}{T}+\frac{1}{3}ln\left(1-e^{-\frac{\Theta_{D}}{T}}\right)-D\left(\frac{\Theta_{D}}{T}\right)\right]$$

in which, k_B_ is the Boltzmann constant, n is the atom number per formula unit and D is the Debye integral given by

(6) $$D\left(y\right)=\frac{3}{y^{3}}\int_{0}^{y}\frac{x^{3}}{e^{x}-1}dx$$

For an isotropic solid, the Debye temperature Θ_D_ can be written as

(7) $$\Theta_{D}=\frac{\hbar }{k_{B}}{\left(6\pi^{2}V^{\frac{1}{2}}n\right)}^{\frac{1}{3}}f\left(\delta \right)\sqrt{\frac{B_{S}}{M}}$$

where M is the molecular mass of Ti_2_MnAl per formula unit and B_S_ is the static bulk modulus, which can be approximated by the static compressibility and given by

(8) $$B_{S}=V\left[\frac{d^{2}E\left(V\right)}{dV^{2}}\right]$$

and δ is the Poisson ratio and f(δ) is expressed by

(9) $$f\left(\delta \right)={\left\{3{\left[2{\left(\frac{2}{3}\frac{1+\delta }{1-2\delta }\right)}^{\frac{3}{2}}+{\left(\frac{1}{3}\frac{1+\delta }{1-\delta }\right)}^{\frac{3}{2}}\right]}^{-1}\right\}}^{\frac{1}{3}}$$

After all, the non-equilibrium Gibbs free energy can be minimized in terms of volume as follow:

(10) $${\left(\frac{\partial G(V;\;P,\;T)}{\partial V}\right)}_{P,T}=0$$

## 3. Results and Discussion

### 3.1. Electronic, Magnetic and Spin-Gapless Behaviors

**Figure 3:**
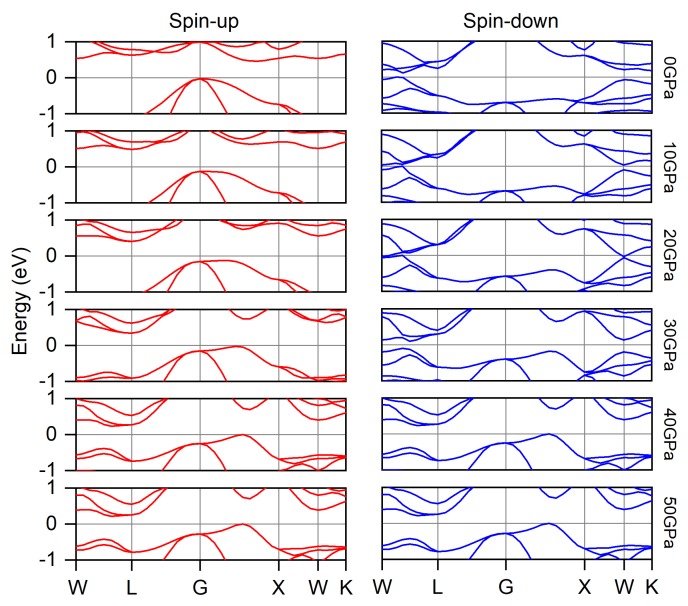
The electronic band structure of Ti_2_MnAl compound around the Fermi energy level (0eV) under different pressure.

**Figure 4:**
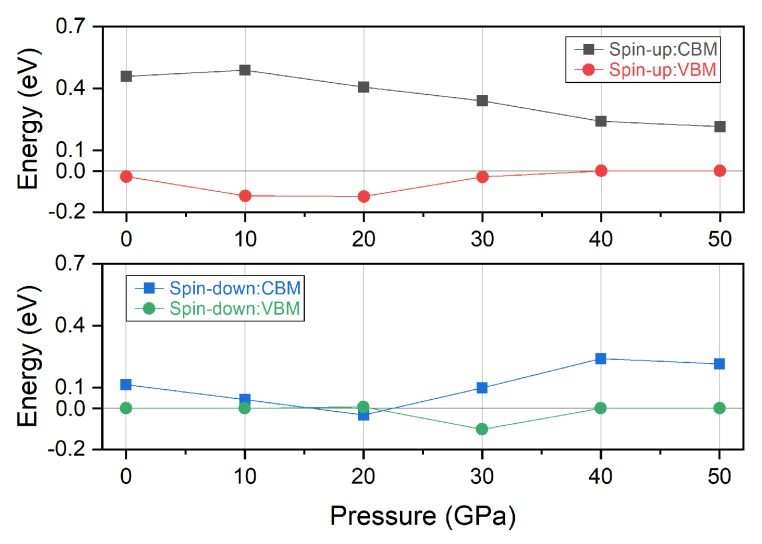
The valence band maximum (VBM) and conduction band minimum (CBM) of Ti_2_MnAl compound in the spin-up and spin-down channels under different pressure.

After we successfully obtain the lattice constants at zero pressure and different external pressure, we calculate the corresponding electronic and magnetic properties of Ti_2_MnAl compound. Figure 3 displays the electronic band structure in spin-up and spin-down channels separately and Figure 4 summarizes the variation of the valence band maximum (VBM) and conduction band minimum (CBM) under different pressure. It can be seen clearly from the band structure that the Fermi energy level (0 eV in the figure) always locates inside the band gap in the spin-up channel within the currently applied pressure range from 0 GPa to 50 GPa. The band gap in the spin-up channel at zero pressure is 0.48 eV and increases at the initial pressure increase to 20 GPa and then decreases quickly with further pressure increase to 50 GPa. While for the spin-down channel, the band gap is 0.11 eV at null pressure and it is much smaller than that in the spin-up channel. The Fermi energy level is still positioned inside this small band gap. Thus, the Ti_2_MnAl compound behaves like spin gapless or nearly spin-gapless semiconductor [41]. With increasing pressure, the conduction band minimum in the spin-down channel moves downwards firstly to below the Fermi level at 20 GPa, leading to the vanish of the band gap, and then ascends again and restore the semiconductor band structure. However, the valence band maximum in the spin-down channel touches the Fermi energy level and almost does not change with pressure variation except at 30 GPa. These behaviors of the band structure variation with pressure or lattice change have a very good agreement with another study [41].

**Figure 5:**
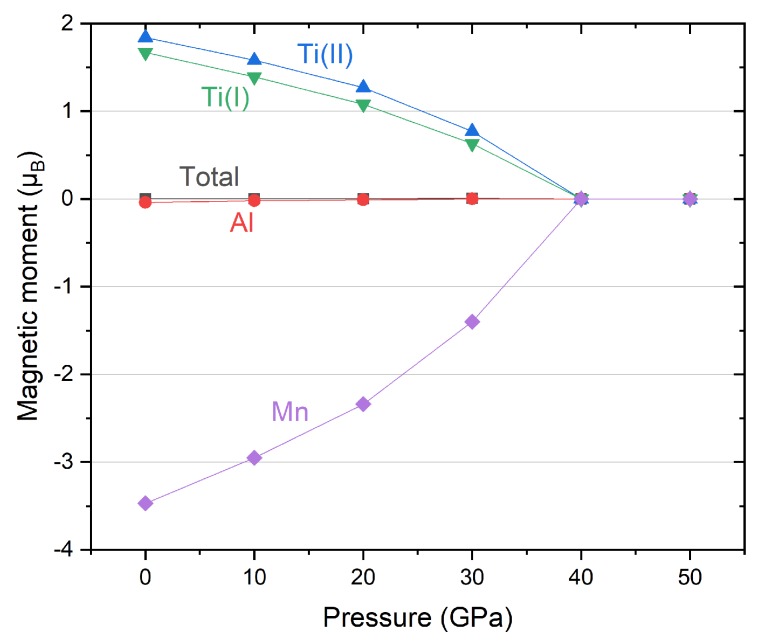
Total and atom-resolved magnetic moments of Ti_2_MnAl compound under different pressure. Atomic site is referred as the crystal structure in Figure 1.

We further calculate the change of the total magnetic moment (TMM) and the atom-resolved magnetic moment (AMM) with pressure change from 0 to 50 GPa and the result is plotted in Figure 5. The magnetic moments of Ti atoms at two different Wyckoff position are both positive initially and also very close to each other. Both of them decrease with pressure increase until they reach zero at 40 GPa. The same sign of the two Ti atoms mean they have same spin direction. While, the magnetic moment of Mn atom is negative and it increases to 0 at 40 GPa. The minus sign indicates the spin direction of Mn atom is antiparallel with the two Ti atoms. Compared with Ti and Mn, the absolute magnetic moment of Al atom is very small and can be negligible. Consequently, Ti and Mn atoms mainly contribute to the total magnetic moment of Ti_2_MnAl. Through the pressure variation, the total magnetic moment is kept at integral value of 0 µ_B_, which is a typical characteristic of the Heusler compound. The Ti_2_MnAl per unit formula has 18 total valence electrons (Z_t_) and the total magnetic moment (M_t_) obeys the Slater-Pauling rule [32,42], M_t_ = Z_t_ − 18. Besides, this zero magnetic moment behavior is also called fully compensated ferromagnetism and it is even more useful than normal half metals since it does not generate stray flux and thus has smaller energy consumption [43]. From the variation of the magnetic moment with pressure, we found that the magnetic property of Ti_2_MnAl is very sensitive to pressure and it change from ferromagnetic to non-magnetic at 40 GPa, which is also confirmed by the overlap of the two total energy curves in Figure 1 at small lattice constant part.

**Figure 6:**
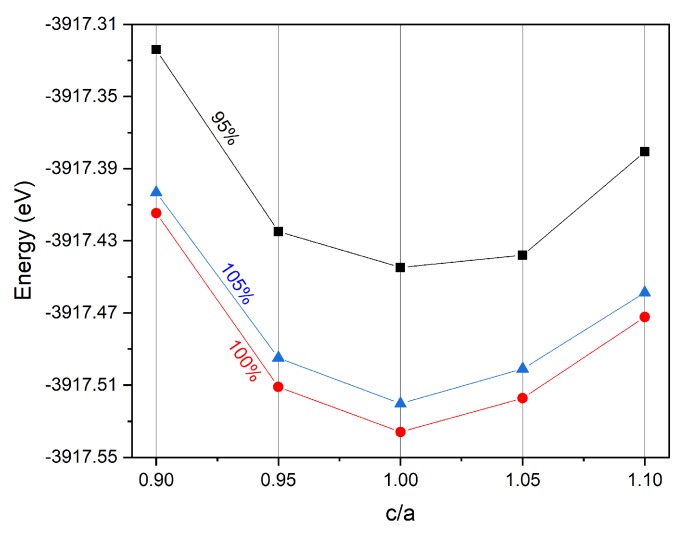
The total energy of Ti_2_MnAl compound with different unit cell volume under tetragonal deformation of different c/a ratio.

In addition, we also examined the tetragonal distortion and its effect on the physical properties of Ti_2_MnAl. Figure 6 shows the total energy against different c/a ratio from 0.9 to 1.1 by keeping the unit cell volume as 95%, 100% and 105% of the equilibrium condition. It can be clearly seen that the total energy with cubic structure (c/a = 1) is always smaller than the tetragonal distorted structure at three different unit cell volume percentages, indicating the tetragonal structure is less stable than the cubic one. Furthermore, the 100% volume has the lowest total energy than the other two under the whole c/a ratio range, which means the equilibrium condition of 100% volume has the most stable structure with the lowest energy and coincides with Figure 1 by comparing the lattice constant variation as the volume change.

**Figure 7:**
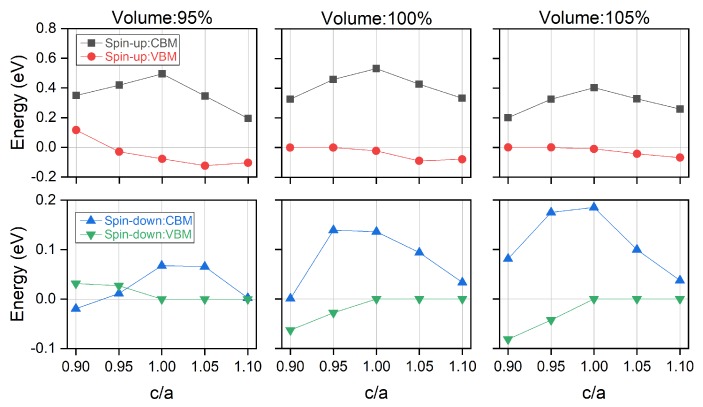
The conduction band minimum (CBM) and valence band maximum (VBM) in spin-up and spin-down directions of Ti_2_MnAl compound with different unit cell volume under tetragonal deformation of different c/a ratio. Please note that the vertical axis has different scales in the two spin directions.

**Figure 8:**
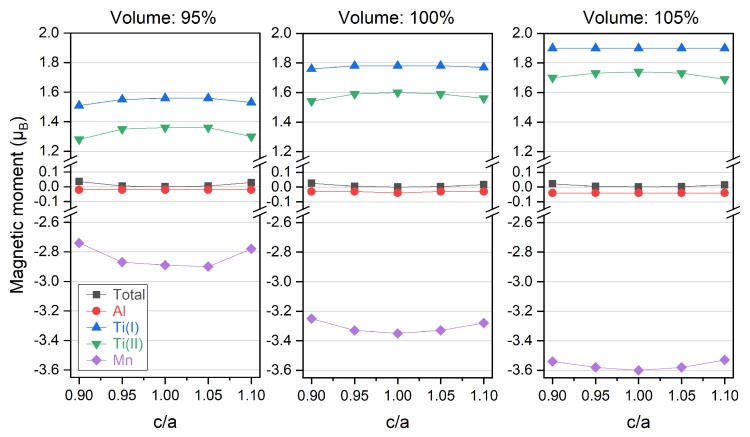
Total and atom-resolved magnetic moments of Ti_2_MnAl compound with different unit cell volume under tetragonal deformation of different c/a ratio. Atomic site is referred as the crystal structure in Figure 1.

With different unit cell volume and under different c/a ratio, we further investigate their effect on the electronic and magnetic properties of Ti_2_MnAl compound and the results are shown in Figure 7 and Figure 8 respectively. It can be found that changing c/a ratio from 1 mostly decreases the conduction band minimum in both spin-up and spin-down directions. While for the valence band maximum, there are different behaviors: it continues to decrease with c/a ratio increase at all three different volume percentages for the spin-up channel; whereas, it decreases at 95% volume yet increase at 100% and 105% percentages in the spin-down channel. It should be pointed out that the spin-gapless feature of Ti_2_MnAl is reflected as the very small band gap in spin-down direction compared with the much larger band gap in the spin-up direction. In addition, this spin-gapless property is only destroyed when c/a ratio is lower than 1 at 95% volume since the conduction band minimum or the valence band maximum in either spin-up or spin-down direction crosses the Fermi energy level. More importantly, a perfect spin-gapless band structure is found at c/a equal to 1.1 at 95% unit cell volume since the valence band maximum and the conduction band minimum touch each other exactly at the Fermi level in the spin-down direction and there is a band gap in the spin-up direction around the Fermi level.

In Figure 8, it is shown the total and atom-resolved magnetic moments vary with c/a ratio under different unit cell volume percentage. It can be observed that tetragonal distortion from the cubic structure always decreases the magnetic moments of the Ti atoms at two sites but increases that of Mn atom, leading to a compensating effect on the total magnetic moment which is almost unchanged and kept at 0 µ_B_. Increasing the unit cell percentage from 95% to 105% results into the stronger variation of the atom-resolved magnetic moments, which matches the trend found in Figure 5 as the absolute atom-resolved magnetic moments decrease with smaller lattice constants at higher pressure values. Overall, the total magnetic moment of Ti_2_MnAl compound shows good stability against the tetragonal distortion with c/a ratio from 0.9 to 1.1.

### 3.2. Mechanic Property and Dynamic Stability

**Figure 9:**
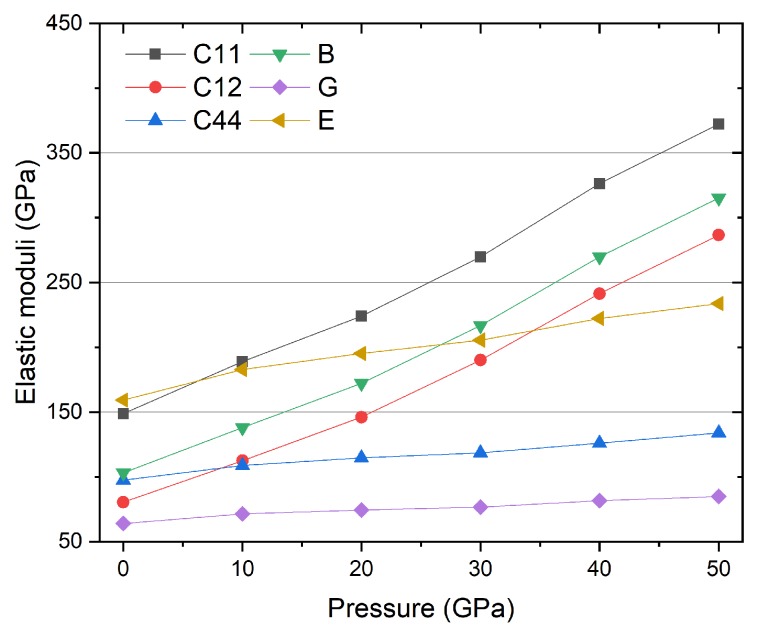
The elastic constants C_ij_, bulk modulus B, shear modulus G and Young’s modulus E of Ti_2_MnAl compound under different pressure.

To further investigate the mechanical property and structural stability of Ti_2_MnAl compound, the elastic constants and several moduli under the pressure range from 0 to 50 GPa are calculated as described in Section 2.3 and the results are listed in Table 1 and plotted in Figure 9. The Pugh’s ratio B/G is also calculated and included in the table. We can see that the three independent elastic constants all increase with pressure, yet they show different change rate. C_11_ and C_12_ have much larger variation with pressure than C_44_. If a cubic structure crystal is mechanically stable, it should satisfy the generalized Born-Huang elastic stability criteria [44,45]:

(11) $$C_{11}>B>C_{12},\;C_{11}+2C_{12}>0,\;C_{44}>0$$

It is found that all the Ti_2_MnAl compound verifies these conditions and thus is mechanically stable through the whole pressure range from 0 to 50 GPa. Next, the bulk modulus, shear modulus and Young’s modulus are studied, which in general define the resistance to shape and volume change. In Figure 9, we can see they all show a modest increase at higher pressure and, in particular, the bulk modulus has a very strong increase than the other two, meaning that the Ti_2_MnAl compound becomes stronger and more difficult to be compressed at high pressure. The Young’s modulus is often applied to measure the stiffness of solids and it becomes larger at high pressure meaning the material gets stiffer. The Pugh’s ratio B/G is equal to 1.61 at null pressure, which is smaller than 1.75 and implies that the compound is brittle based on the Pugh’s criteria. However, this ratio immediately jumps to larger than 1.75 and becomes even larger with pressure increase making the compound more and more ductile. In summary, all the elastic constants and moduli become larger at higher pressure but with different changing rate.

**Figure 10:**
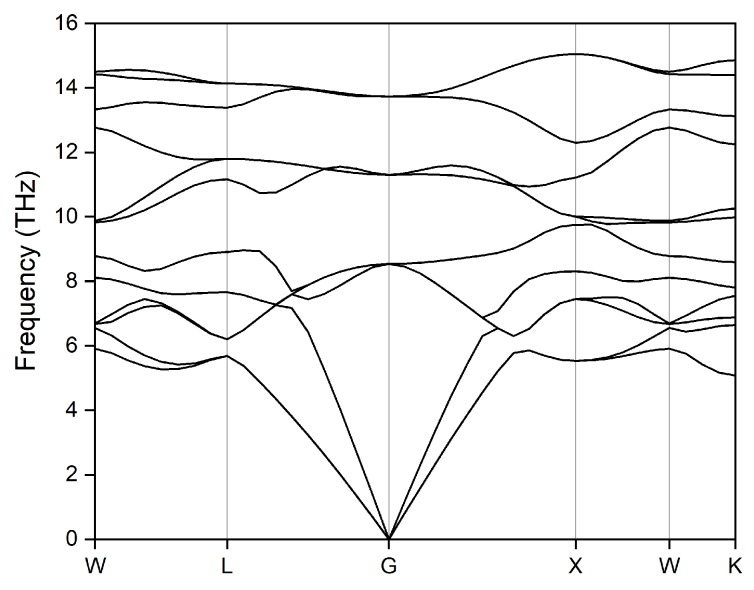
The photon dispersion spectrum of Ti_2_MnAl compound calculated at 50 GPa.

Dynamic stability is another very important property for a material and it can be examined by the phonon modes. To understand this of Ti_2_MnAl at high pressure, we further calculated the phonon dispersion spectrum in the whole Brillouin zone along the W-L-G-X-W-K directions at 50 GPa and the result is displayed in Figure 10. It is clearly shown that there are no imaginary frequencies in the phonon dispersion curve, which reveals that Ti_2_MnAl is dynamically stable even at so high pressure of 50 GPa.

### 3.3. Thermodynamic Properties

In the end, we also examined the thermodynamic properties of Ti_2_MnAl compound to even extend our knowledge about its specific behavior under higher pressure condition. After the quasi-harmonic Debye model [38,39,40] is applied as described in Section 2.4 and the equation of state can be derived by solving Equation (10). Detailed process can be found in Refs. [38,39,40]. Then, we can obtain the isothermal bulk modulus (B) and the specific heat capacity at constant volume (C_V_) by the following expressions:

(12) $$B\left(P,\;T\right)=V{\left[\frac{\partial^{2}G\left(V;\;P,\;T\right)}{\partial V^{2}}\right]}_{P,T}$$

(13) $$C_{V}=3nk_{B}\left[4D\left(\frac{\Theta_{D}}{T}\right)-\frac{3\frac{\Theta_{D}}{T}}{e^{\frac{\Theta_{D}}{T}}-1}\right]$$

where γ is the Grüneisen constant and α is the thermal expansion coefficient, and they are given by

(14) $$\gamma =\frac{d\;ln\Theta_{D}}{d\;lnV},\;\alpha =\frac{\gamma C_{V}}{B_{T}V}$$

Then we calculated the normalized variation of the primitive cell volume, the thermal expansion coefficient α, heat capacity at constant volume C_V_, Grüneisen constant γ and Debye temperature Θ_D_ with pressure under the same range from 0 to 50 GPa. Besides, we also included the temperature effects from 0 to 1500 K.

**Figure 11:**
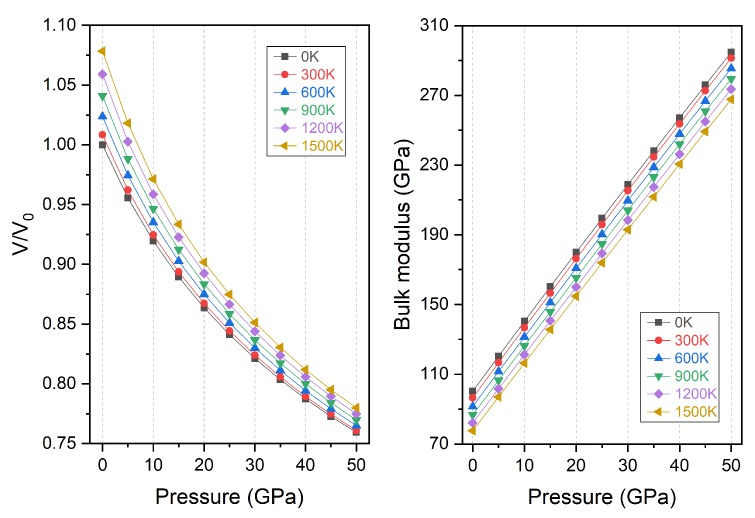
The normalized volume (V/V_0_) and bulk modulus variation with pressure for Ti_2_MnAl compound.

It is shown in Figure 11 that the normalized unit cell volume varies with pressure at different temperature. V_0_ refers to the unit cell volume at equilibrium condition of 0 GPa and 0 K and V the volume at pressure P and temperature T. With pressure increase, the volume decreases for all different temperature series. This is obviously expected because the material is compressed and the lattice is smaller at higher pressure. In particular, for the 0 k condition, the volume variation with pressure is also reflected as the lattice change as shown in Figure 2. However, the relative volume increases with temperature at the same pressure and this is apparently due to thermal expansion. It can also be found that the changing rate of the volume at higher temperature is larger, which shows the Ti_2_MnAl is easier to be compressed at higher temperature. The dependence of bulk modulus with pressure is also displayed in Figure 11. It can be seen that the bulk modulus almost increases linearly with pressure increase at a given temperature and decreases with temperature at a given pressure. Different temperature only makes an offset in the vertical direction at any given pressure. It should be noted that the bulk modulus variation with pressure at 0 K matches with the result of bulk modulus in Figure 9 very well and this coincidence indicates the good conformity of the theoretical analysis from two methods. Meanwhile, we found that the pressure has a much stronger effect on the variation of both the volume and the bulk modulus than temperature.

**Figure 12:**
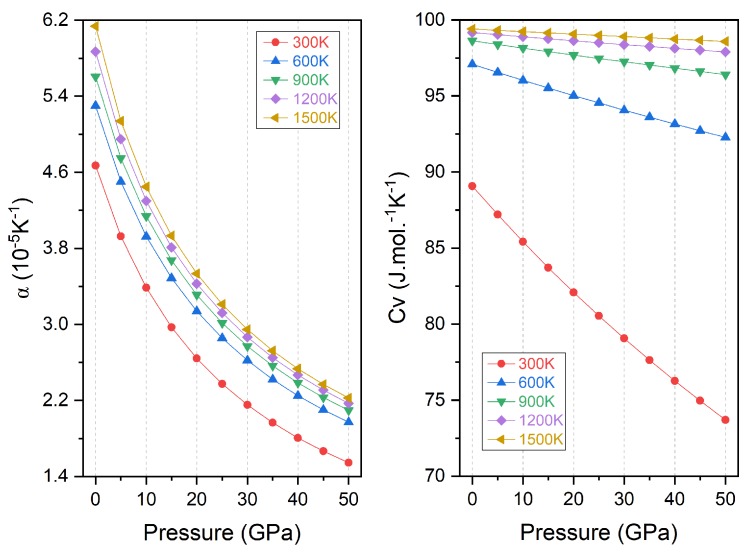
The thermal expansion coefficient (α) and heat capacity at constant volume (C_V_) variation with pressure for Ti_2_MnAl.

The thermal expansion coefficient α is both theoretically and practically significant and is essential for the thermodynamic equation of state. The heat capacity at constant volume C_V_ is another critical thermodynamic parameter because it can provide information about the lattice vibration and transition of solid phase. Their variations with pressure are presented in Figure 12. It is shown that the thermal expansion coefficient decreases steeply with pressure at the initial range from 0 to 20 GPa and slowly at higher pressure. Higher temperature only shifts the curve vertically to the upward direction and thus increases the thermal expansion coefficient. The change rate with pressure is much stronger than that of temperature. At 0 GPa and 300 K, the thermal expansion coefficient is equal to 4.67 × 10${}^{-5}$ K${}^{-1}$. Whereas, the variation of thermal capacity with pressure is very different at different temperature: at low temperature of 300 K, the thermal capacity decreases sharply with pressure increase due to the anharmonic approximation in the Debye model; at higher temperature, this changing rate is strongly depressed and it almost does not change with pressure when the temperature is higher than 800 K. Besides, the heat capacity is expected to converge to the Dulong-Petit limit of 99.774 JMol${}^{-1}$ K${}^{-1}$ at higher temperature, meaning the thermal energy excites all the possible phonon mode. The heat capacity shows opposite change with temperature and pressure and it is equal to 89.08 JMol${}^{-1}$ K${}^{-1}$ at null pressure and 0 K.

**Figure 13:**
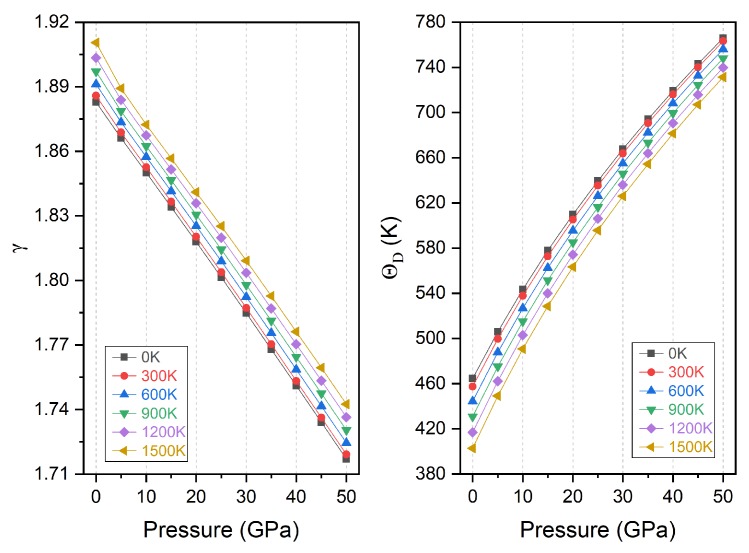
The Grüneisen constant (γ) and Debye temperature (Θ_D_) variation with pressure for Ti_2_MnAl.

Finally, we also studied the evolution of the Grüneisen constant γ and the Debye temperature Θ_D_ with pressure and the results are presented in Figure 13. The Grüneisen constant is very important parameter since it exists in some useful thermodynamic relations. It is clearly shown that the Grüneisen constant has very little change when the temperature is lower than 300 K and then it increases almost linearly with further temperature increase. At a given temperature, it decreases significantly upon compression under high pressure. The Debye temperature is also a fundamental parameter for a material because it is related with many important physical properties, including specific heat, melting temperature and so on. In particular, for the lattice vibration, when the temperature is below the Debye temperature, the main lattice vibration is from acoustic part and the quantum mechanical effect contributes only to the thermodynamic properties. We note from Figure 13 that the Debye temperature is nearly constant from 0 to 300 K and then decreases with higher temperature. For a given temperature, it increases drastically with pressure and at higher pressure the variation from temperature becomes less pronounced. Overall, we found that the pressure has a much stronger effect on the Grüneisen constant and Debye temperature than temperature. The calculated Grüneisen constant and the Debye temperature for Ti_2_MnAl compound at zero pressure and 300 K are 1.88 and 457.37 K respectively.

## 4. Conclusions

In this work, we have extensively and systematically studied the structural, electronic, magnetic, mechanical and thermodynamic properties of the fully compensated spin-gapless inverse Heusler Ti_2_MnAl compound under pressure condition by employing the first-principles calculation based on density functional theory and the quasi-harmonic Debye model. The obtained results for lattice constant, spin-gapless semiconductor behavior and fully compensated ferromagnetism at ground state without external pressure have a good agreement with previous studies. By applying pressure from 0 to 50 GPa, the lattice constants decrease and the total energies increases. For the spin-gapless behavior, it is destroyed at pressure of 20 GPa and then restored again with higher pressure. For the magnetic properties, the atom-resolved magnetic moments change stronger with pressure variation. However, the fully compensated ferromagnetism shows good resistance upon pressure to 30 GPa and becomes non-magnetism when the pressure is larger. Thus, the fully compensated ferromagnetism and spin-gapless behavior have been maintained only at lower pressure range from 0 to 10 Gpa. Besides, under the tetragonal distortion of c/a ratio from 0.9 to 1.1 at three unit cell volume percentages: 95%, 100% and 105%, only the spin-gapless behavior is destroyed when c/a is less than 1 at 95% volume and the fully compensated ferromagnetism is always kept. In terms of the mechanical property, three independent elastic constants and various moduli have been calculated, and they all shown a moderate increase with pressure. The mechanical stability is examined with the generalized Born-Huang elastic stability criteria and it is found Ti_2_MnAl is mechanically stable under the whole studied pressure range. The Pugh’s ratio is equal to 1.61 at null pressure, indicating that the material is brittle, and then jumps to higher than 1.75 with larger pressure leading to the ductile transformation. Besides, the dynamic stability is also investigated by the phonon dispersion spectrum and results show Ti_2_MnAl is dynamically stable even at very high pressure of 50 GPa. In the end, several thermodynamic parameters were also studies under pressure condition with the quasi-harmonic Debye model, including the normalized volume, thermal expansion coefficient, heat capacity at constant volume, Grüneisen constant and Debye temperature. It is found that temperature and pressure have the same or opposite effects dependent on the studied parameter, and they also show quite different influence intensity. Up to now, there is still no experimental or theoretical values for the thermodynamic properties of Ti_2_MnAl and, thus, our result can serve as a helpful reference for the future work.

## Figures and Tables

**Table 1:** The elastic constants C_ij_, bulk modulus B, shear modulus G, Young’s modulus E and Pugh’s ratio B/G of Ti_2_MnAl compound under different pressure.

P	C_11_	C_12_	C_44_	B	G	E	B/G
(GPa)	(GPa)	(GPa)	(GPa)	(GPa)	(GPa)	(GPa)	
0	148.79	80.46	97.49	103.23	64.07	159.27	1.61
10	188.84	112.49	108.70	137.94	71.50	182.90	1.93
20	224.07	146.10	114.62	172.09	74.45	195.21	2.31
30	269.74	190.16	118.48	216.69	76.57	205.51	2.83
40	326.34	241.45	126.10	269.75	81.57	222.31	3.31
50	372.21	286.61	133.91	315.14	84.90	233.71	3.71
